# Cognitive appraisals linking dispositional mindfulness to athletes' emotions: a multi-states theory approach

**DOI:** 10.3389/fspor.2024.1521613

**Published:** 2024-12-18

**Authors:** Claudio Robazza, Francesca Vitali, Laura Bortoli, Montse C. Ruiz

**Affiliations:** ^1^ BIND-Behavioral Imaging and Neural Dynamics Center, Department of Medicine and Aging Sciences, “G. d’Annunzio” University of Chieti-Pescara, Chieti, Italy; ^2^ Department of Neurosciences, Biomedicine and Movement Sciences, University of Verona, Verona, Italy; ^3^ “G. d’Annunzio” University of Chieti-Pescara, Chieti, Italy; ^4^ Faculty of Sport and Health Sciences, University of Jyväskylä, Jyväskylä, Finland

**Keywords:** mindful awareness, refocusing, challenge & threat, functional emotions, psychobiosocial experiences

## Abstract

**Purpose:**

The aim of the study was to examine the relationship between dispositional mindfulness, cognitive appraisals, emotions, and psychobiosocial experiences in athletes within the framework of multi-states (MuSt) theory.

**Method:**

A convenience sample of 334 Italian athletes (188 men and 146 women), aged 18–48 years (*M* = 24.77, *SD* = 7.26) and involved in individual or team sports, were recruited for the study. Athletes were assessed individually or in small groups before regular practice sessions.

**Results:**

Path analysis showed positive indirect effects via challenge appraisal from mindful awareness and refocusing to excitement, happiness, and psychobiosocial experiences, and negative indirect effects to anxiety and dejection. Positive indirect effects were observed via threat appraisal from mindful awareness and refocusing to happiness and psychobiosocial experiences, and negative indirect effects to anxiety, dejection, and anger.

**Conclusions:**

The results highlight the impact of mindful awareness, refocusing, and cognitive appraisals on athletes' emotional and psychobiosocial experiences. Overall findings support MuSt theoretical foundation and suggest that mindfulness may help athletes view challenges as opportunities to express their potential by triggering pleasant emotions and functional psychobiosocial experiences. From an applied perspective, the findings support the use of mindfulness practice in the development of programs to promote athletes' challenge appraisals, pleasant and functional emotional experiences, which may enhance their performance.

## 1 Introduction

There is growing interest in sport psychology in understanding the factors that influence athletic performance, particularly the cognitive and emotional processes that occur under competitive pressure (1–3). Understanding how athletes manage stress, regulate their emotions, and maintain focus is fundamental to help them achieve optimal performance. This knowledge not only provides insight into the psychological mechanisms underlying high achievements, but also informs the development of interventions designed to enhance athletes' mental conditions and their overall well-being (4). One key area of exploration in this context is the relationship between cognitive appraisals, emotional experiences, and performance outcomes. Cognitive appraisal theories [see (5)] suggest that the way in which athletes interpret and evaluate stressful situations significantly influences their emotional responses and subsequent achievements. Furthermore, there is an increasing interest in how mindfulness practice, which emphasizes present-moment awareness and acceptance, might interact with psychological processes to shape emotional experiences and support optimal performance (6). Given the complex and dynamic nature of these mental processes, it is important to examine how mindfulness, cognitive appraisals, and emotional experiences interact. Investigating this topic can offer insights into how athletes can better manage the psychological demands of competitions, eventually leading to enhanced performance and well-being.

Rooted in ancient Buddhist practices aimed at reducing suffering and enhancing well-being, mindfulness has been adapted into Western psychology mainly through the work of Jon Kabat-Zinn, who developed a mindfulness-based stress reduction program initially designed to treat chronic pain in terminally ill patients (7). Kabat-Zinn applied mindfulness techniques to help elite athletes, emphasizing its potential to enhance performance by fostering a present-moment awareness that is both purposeful and nonjudgmental (8). Mindfulness is fundamentally about paying attention to the present moment in an intentional, nonjudgmental way, allowing individuals to engage fully with their current experiences, including thoughts, emotions, and bodily sensations. This practice contrasts with traditional emotion regulation strategies, which often involve efforts to modify or suppress emotions (9). Instead, within mindfulness practice people are encouraged to observe and accept their internal experiences as they occur, without attempting to alter or avoid them (10). In sport, this approach has been used to help athletes manage stress, maintain focus, and avoid becoming overwhelmed by dysfunctional emotions, which can be detrimental for performance.

Mindfulness has gained considerable attention in sport for its ability to enhance athletic performance and promote mental well-being (11). Mindfulness-based programs aim to help athletes cultivate awareness of their internal experiences, enabling them to stay focused and effectively manage competitive stress. Unlike traditional behavioral interventions that emphasize control over negative thoughts and feelings, mindfulness encourages acceptance of all experiences, whether positive or negative, fostering emotional resilience and reducing psychological distress. This practice helps athletes maintain an optimal mental state by redirecting their focus from disruptive thoughts back to performance-related tasks. Mindfulness also promotes cognitive *defusion*—a state where thoughts and feelings are recognized as transient and separate from the self—allowing athletes to avoid becoming entangled in their emotions (12). This mental presence, where thoughts and emotions are acknowledged without judgment, is crucial for maintaining focus and improving performance. Based on Gardner and Moore's mindfulness-acceptance-commitment model in sport, Thienot et al. (13) developed the Mindfulness Inventory for Sport (MIS) Scale to assess trait mindfulness. This tool comprises an awareness component (i.e., noticing disruptive thoughts, emotions, and bodily sensations in the stream of consciousness), an acceptance, non-judgmental component (i.e., embracing the presence of disruptive stimuli without self-judgment), and a refocusing component (i.e., redirecting attention from disruptive stimuli to beneficial, goal-oriented cues).

Several reviews of the literature highlight the benefits of mindfulness in sport, including improved awareness, attention, emotions, and emotional regulation (14, 15). Sappington and Longshore (16) found preliminary evidence that mindfulness-based interventions enhance athletic performance, while Bühlmayer et al. (17) noted positive effects on psychological and physiological variables, particularly in precision sports. Other reviews suggest that mindfulness can reduce competitive anxiety, prevent injuries, and boost confidence (18, 19). Furthermore, mindfulness may enhance performance monitoring abilities, crucial for error detection and adjustment (20). Overall, mindfulness-based interventions offer significant benefits for athletes, including improved attention control, better emotion regulation, reduced psychological distress, enhanced performance, and potential health benefits, such as burnout reduction and injury prevention.

Regarding emotional experiences, dispositional mindfulness has been linked to athletes' flow states, which are characterized by complete immersion and focus on the task (21). Moreover, a mindfulness-based stress reduction program was found to improve functional psychobiosocial states in a sample of athletes (22). Psychobiosocial experiences encompass a spectrum of emotional and non-emotional aspects of subjective feelings related to past, present, and anticipated future performances (23). These experiences entail psychological (e.g., emotional, confidence, cognitive, motivational), biological (bodily, motor-behavioral), and social (e.g., communicative, social support) components. Research has found that dispositional mindfulness mediates the relationship between personality traits such as conscientiousness and emotional stability with psychobiosocial states (24). Path analysis revealed a significant positive indirect effect of conscientiousness on functional psychobiosocial states through awareness and refocusing dimensions, while emotional stability showed a positive indirect effect via refocusing. These studies highlight the pivotal role of mindfulness in enhancing the emotional experiences of athletes.

In their transactional model of stress, Lazarus and Folkman (25) argued that individual responses to stress involve a dynamic interaction between primary and secondary cognitive appraisals. Primary appraisals involve assessing the significance of an encounter, while secondary appraisals evaluate the potential to mitigate harm or maximize benefits. Individual evaluations determine whether an individual adopts a “challenge” state, where perceived resources meet or exceed demands, or a “threat” state, where demands outweigh resources. These states exist on a continuum, allowing for varying degrees of challenge or threat in response to performance demands, which may be cognitive, affective, or physical in nature. These appraisals collectively shape the intensity and type of emotions experienced by the person. Building on this framework, Blascovich and colleagues [e.g., (26, 27)] developed the biopsychosocial model of challenge and threat, which outlines how individuals assess demands and resources in motivated performance situations influencing their physiological responses. In line with this model, challenge and threat states only occur in motivated performance situations, where individuals are driven to excel and are evaluated on their performance, resulting in different patterns of cardiovascular responses (26). For a challenge or threat state to emerge, the situation must be perceived as both goal-relevant and evaluative. Perceptions of personal resources are key in this evaluative process, determining whether an individual perceives a situation as a challenge or a threat.

As an extension of the biopsychosocial model, Jones et al. (28) proposed a theory of challenge and threat states in athletes, which was subsequently revised by Meijen et al. (29). This theory posits that challenge states are associated with both pleasant and unpleasant emotions, while threat states are predominantly linked to unpleasant emotions. For example, an athlete in a challenge state may experience a mix of excitement and nervousness, whereas a threat state might only evoke anxiety. The interpretation of these emotions also differs; in a challenge state, nervousness, for instance is seen as facilitating performance, while in a threat state, it is viewed as detrimental. This suggests that in a challenge state, unpleasant emotions can be counterbalanced by pleasant ones or reinterpreted as beneficial (5).

Empirical evidence supports the idea that challenge states are associated with more functional emotions, better coping expectancies, and lower anxiety levels. These states are linked to better performance outcomes than threat states (30, 31). A systematic review by Hase et al. (32) reinforced these findings, indicating that challenge states consistently lead to better performance across various tasks and study designs [see (5)]. In their meta-analysis, Behnke and Kaczmarek (33) found stable effects linking cardiovascular markers of challenge and threat states to successful performance.

In the current study we examined the relationship between mindfulness, cognitive appraisals, and emotional experiences within the framework of multi-states (MuSt) theory (34), which shares similarities with the transactional model of stress (25) and the theory of challenge and threat states in athletes (28, 29). MuSt theory aims to offer a comprehensive framework for understanding the variety of performance-related experiences of athletes during training and competition, as well as predicting their performance outcomes. According to MuSt theory, performance is a dynamic and multifaceted process arising from the interactions between the individual, task, and environment, which act as antecedents of athletes' subjective experiences. Researchers have examined individual dispositional antecedents of psychobiosocial experiences and the mediating role of cognitive appraisals within the framework of MuSt theory. For instance, empirical evidence showed that in athletes from different sports, perfectionistic strivings positively predicted functional states through challenge appraisal, while perfectionistic concerns positively predicted dysfunctional states via threat appraisal (35). Perfectionism was also studied in soccer referees (36), with findings showing positive indirect effects of both self-oriented and socially prescribed perfectionism on self-evaluated performance via challenge appraisal and psychobiosocial experiences. Moreover, in medalist kickboxers participating in the WAKO World Kickboxing Championship, a positive indirect link was found from self-confidence to self-evaluated performance via challenge appraisal and psychobiosocial experiences, while negative indirect links were identified from worry and concentration disruption to self-evaluated performance via threat appraisal and psychobiosocial experiences (37).

These studies collectively support the underpinnings of MuSt theory. However, the relationship between dispositional mindfulness and psychobiosocial experiences and the potential mediating role of cognitive appraisals have not yet been examined. A related line of research in work settings explored similar mechanisms based on Lazarus and Folkman's (25) transactional model of stress. Jamieson et al. (38) and Toniolo-Barrios and ten Brummelhuis (39) noted that, despite a substantial body of literature demonstrating the stress-reducing effects of mindfulness, the underlying psychological mechanisms remain largely unexplored. They further argued that many studies have been conducted without a strong theoretical foundation, creating a gap in both scholarly and practical understanding of mindfulness. This gap hinders researchers from fully understanding the psychological processes involved in the stress-reducing effects of mindfulness and limits practitioners' ability to develop more effective interventions. In response to this gap, Jamieson et al. (38) applied the transactional model of stress to better understand the beneficial effects of mindfulness on employees. Their findings showed that mindfulness led to increased challenge appraisal and reduced threat appraisal, which, in turn, promoted a more positive state characterized by increased pleasant affect and reduced unpleasant affect. Using the same transactional model of stress, Toniolo-Barrios and ten Brummelhuis (39) showed that mindfulness was associated with reduced work-related stress among employees, primarily through decreased threat appraisal.

**Figure 1:**
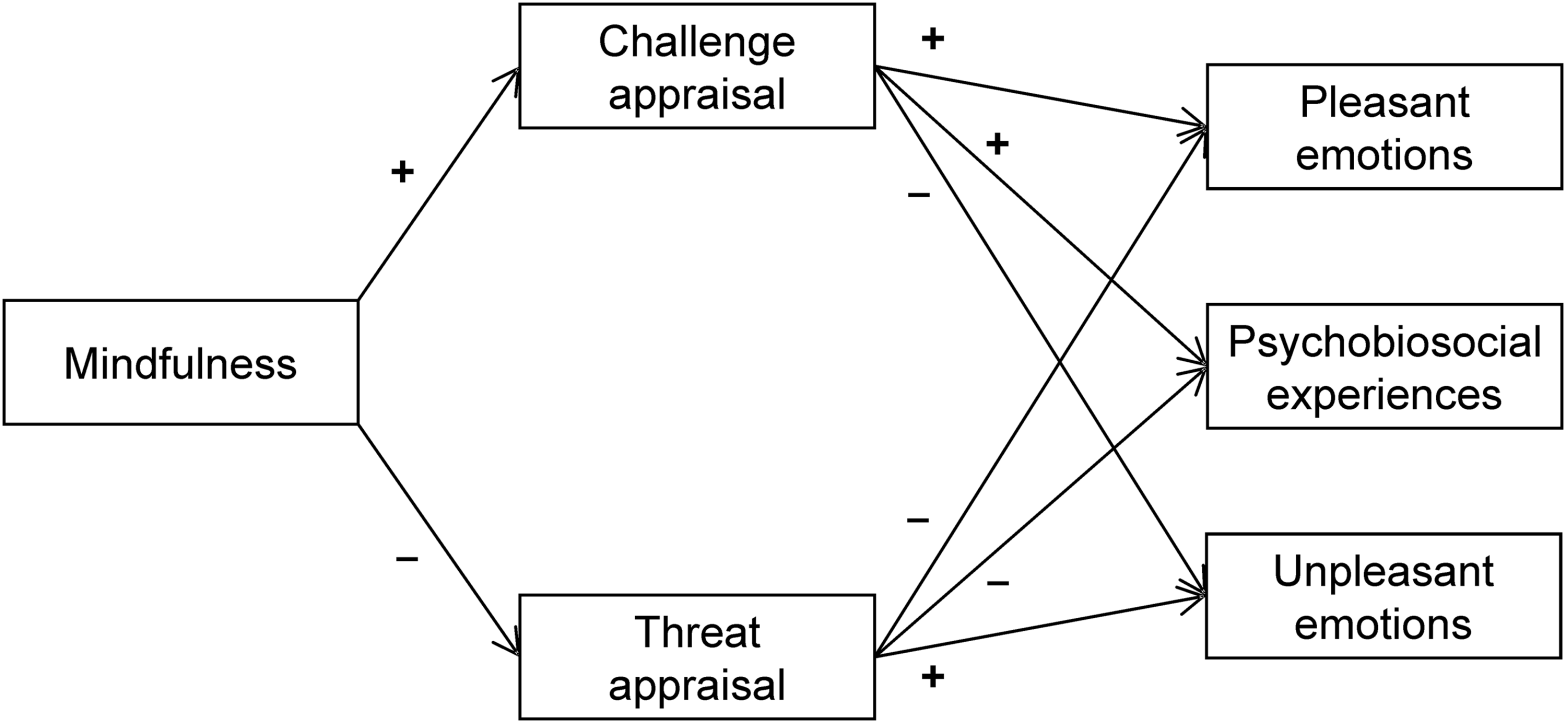
Hypothesized model.

Building on existing line of research and drawing on the foundations of MuSt theory (34), the current study aimed to investigate whether mindfulness influenced athletes' emotions and psychobiosocial experiences via the mediation of cognitive appraisals. Specifically, as depicted in Figure 1, we examined the effects of mindfulness on pleasant emotions, psychobiosocial experiences, and unpleasant emotions through cognitive appraisals. We anticipated that mindfulness and cognitive appraisals would similarly affect both pleasant emotions and psychobiosocial experiences, following previous research indicating that athletes typically report psychobiosocial feelings that are functional for performance in competitive settings (37, 40, 41). Based on this rationale, we expected to find positive relationships between both dispositional mindfulness and challenge appraisal with the experience of pleasant emotions and functional psychobiosocial feelings (22, 24), as well as negative relationships with unpleasant emotions. Conversely, threat appraisal was expected to be positively related to unpleasant emotions and negatively related to pleasant emotions. Beyond these predictions, the novel contribution of this study to the existing literature lies in the mediating effects of cognitive appraisals on the relationship between mindfulness and emotional experiences. To this end, we formulated two hypotheses:

**Hypothesis 1:** Challenge appraisal was expected to positively mediate the relationship between mindfulness and pleasant emotions/psychobiosocial experiences, and negatively mediate the relationship between mindfulness and unpleasant emotions. Considering the antecedent factor, mindfulness may positively influence challenge appraisal, which could potentially increase pleasant emotions and all components of psychobiosocial experiences while decreasing unpleasant emotions (Figure 1).
**Hypothesis 2:** Threat appraisal was expected to negatively mediate the relationship between mindfulness and pleasant emotions/psychobiosocial experiences, and positively mediate the relationship between mindfulness and unpleasant emotions. Therefore, mindfulness may associate with less threat appraisal experience, thereby buffering its negative effect on pleasant emotions and all components of psychobiosocial experiences, as well as its positive impact on unpleasant emotions (Figure 1).

## 2 Method

### 2.1 Participants

To ensure adequate statistical power, a minimum sample size was established based on rule-of-thumb indications (42, 43). A minimum of 10 cases per estimated model parameter is recommended, requiring a sample size of 180 for our model with 18 parameters. Additionally, the minimum sample size for root-mean-square error of approximation (RMSEA) was calculated using Preacher and Coffman's (44) R code (https://cran.r-project.org). With an alpha level of .05, 33 degrees of freedom, a power of .80, and an alternative RMSEA of .08 (indicating acceptable model fit), the suggested sample size was 291. The initial sample consisted of 338 participants from 60 sporting clubs. After excluding 4 outliers, the final sample comprised 334 participants (188 men and 146 women), aged 18–48 years (*M* = 24.77, *SD* = 7.26). Participants were recruited from individual sports (*n* = 219; e.g., fencing, gymnastics, martial arts, swimming, tennis, and track & field) and team sports (*n* = 115; e.g., basketball, futsal, rugby, soccer, and volleyball). The mean years of sport participation were 12.23 (*SD* = 7.29) for men and 10.74 (*SD* = 6.66) for women at regional (49.7%), national (37.7%), or international level (12.6%).

### 2.2 Measures

To reduce both the time and psychological burden involved in gathering data, thereby promoting accurate and reliable responses, we carefully identified key items to capture constructs from mindfulness and cognitive appraisal measures. Therefore, three items were selected for each subscale of each instrument to ensure relatively short measures that are easily applicable in the sport context, while still providing coverage of the theoretical domain of the construct (42). This process was based on a consensus developed through a triangulation method among the researchers involved in the study. The joint process of verification and selection of items aimed to ensure that the chosen items accurately reflected the fundamental constructs being studied. Moreover, we merged items representing five distinct emotions into composite items, each indicative of a specific emotional state. The same procedure was applied to the psychobiosocial experiences measure, where items representing 12 distinct modalities were merged into composite items. This approach of using single items aligns with the guidelines and practices observed in existing research [e.g., (45, 46)], highlighting the practical benefits of this data collection approach.

#### 2.2.1 Mindfulness

To assess dispositional mindfulness, we used the Mindfulness Inventory for Sport (MIS) Scale (13), a context-specific tool designed to measure trait mindfulness in sport settings. This scale includes 15 items in three subscales: Awareness (e.g., “I am aware of the thoughts that are passing through my mind”), Non-judgmental attitude (e.g., “When I become aware that I am not focusing on my own performance, I blame myself for being distracted”), and Refocusing (e.g., “When I become aware that I am tense, I am able to quickly bring my attention back to what I should focus on”). Three items were selected for each subscale. Athletes rated how much each statement reflected their usual sport experience during competition on a scale from 1 (*not at all*) to 6 (*very much*). Like the original English version, the items in the Non-judgmental attitude subscale were reverse scored, and the mean of the items was calculated for each subscale. The factor structure of the MIS scale, adapted to the Italian language, was supported by previous research (24), which demonstrated acceptable internal consistency (omega coefficients) for Awareness (.874), Non-judgmental attitude (.751), and Refocusing (.865).

#### 2.2.2 Cognitive appraisals

Cognitive appraisals to confront an upcoming important competition were assessed using the Challenge and Threat in Sport (CAT-Sport) scale (47). This measure consists of two subscales, Challenge and Threat, which assess how athletes perceive their resources and abilities in relation to the demands of a situation. The Challenge subscale reflects an athlete's belief in their potential for success (e.g., “I anticipate achieving success rather than experiencing failure”), whereas the Threat subscale captures the perception that the demands of a situation surpass their available resources (e.g., “I feel this task is a threat”). Three items were used for each scale. Responses were rated on a 6-point Likert scale, ranging from 1 (*totally disagree*) to 6 (*totally agree*). Confirmatory factor analysis supported the 2-factor model, with good internal consistency and criterion validity (47). Following back-translation procedures (48), the CAT-Sport was translated and adapted into Italian language.

#### 2.2.3 Emotions

Emotional states in competitive contexts were assessed using the items of the Sport Emotion Questionnaire [SEQ; (49)]. The SEQ items of each scale were merged into single items to assess five discrete emotional states: Excitement (“exhilarated, excited, enthusiastic, energetic”), Happiness (“pleased, joyful, happy, cheerful”), Anxiety (“uneasy, tense, nervous, apprehensive, anxious”), Dejection (“upset, sad, unhappy, disappointed, dejected”), and Anger (“irritated, furious, annoyed, angry”). Participants were asked to reflect on the intensity of each emotional state they anticipated experiencing before a forthcoming important competition, and assess themselves on a 5-point scale, ranging from 0 (*not at all*) to 4 (*extremely*). The Italian version of the SEQ demonstrated a satisfactory factor structure and reliability, with Cronbach's alpha values between .741 and .863, and composite reliability values between .742 and .864 (50).

#### 2.2.4 Psychobiosocial experiences

Psychobiosocial experiences were assessed through the 12-item stimulus list for individualized profiling (see Supplementary Data Sheet 1). The list is based on the multidimensional profiling developed by Ruiz et al. (34) and the Psychobiosocial Experience Semantic Differential scale for sport (PESD-Sport; (40)]. The items were arranged in a semantic differential format. Each bipolar item represented 12 modalities through 4–5 adjectives each: emotion, confidence, anxiety, assertiveness, cognitive, motivational, and volitional (psychological component); bodily-somatic, motor-behavioral, and operational (biological component); and communicative and social support (social component). Each item consisted of bipolar adjective pairs, with dysfunctional descriptors on the left extremity and their functional antonyms on the right of a Liker-type scale (e.g., “Dejected, unhappy, sad, distressed” vs. “Enthusiastic, happy, joyful, cheerful”; “Physically fatigued, tired, drained, drowsy” vs. “Physically vigorous, charged, reactive, energetic”). Participants were instructed to reflect on their anticipated emotional experiences prior to an upcoming important competition and, based on its potential impact on performance, rate each descriptor on a Liker-type scale from 4 (*very much*) to 1 (*a little*) on the dysfunctional side, or from 1 (*a little*) to 4 (*very much*) on the functional side. A score of 0 (*neither…nor*) was assigned if a descriptor was deemed non-influential. Ratings on the dysfunctional side were then converted to negative scores, resulting in a range of −4 to 4 for each item, with 0 indicating no effect. The PESD-Sport has been previously validated among Italian athletes, showing satisfactory factorial, construct, convergent, discriminant, and nomological validity (40).

### 2.3 Procedure

This study adhered to the Declaration of Helsinki and received ethical approval from the local university ethics committee. Sport managers and coaches of clubs in central Italy were contacted and informed about the study purpose to gain permission to approach athletes. Eligibility criteria included: training at least twice weekly, participating in regular competitions during the season at a regional level or higher, and being 18 years or older. Prior to participation, athletes were informed about the voluntary nature of the study, their right to withdraw at any time without consequences, and the confidentiality of their responses. They were briefed on the general purpose of the study and provided with instructions emphasizing that there were no correct or incorrect answers. Written informed consent was obtained from all participants. Assessment sessions were conducted individually or in small groups (maximum five participants) in a quiet location before regular practice sessions. An investigator ensured that participants understood the instructions and completed all items of the measures. After providing informed consent, the athletes completed the survey comprising sociodemographic questions (e.g., age, gender, years of practice) and all measures. The assessment took approximately 20 min to complete.

### 2.4 Data analysis

The dataset was screened to identify potential univariate and multivariate outliers on the mean total scores of Awareness, Non-judgmental attitude, Refocusing, Challenge appraisal, Threat appraisal, and Psychobiosocial experiences, as well as the mean scores of single-item measures of Excitement, Happiness, Anxiety, Dejection, and Anger. Normality and multicollinearity assumptions were examined (42). Descriptive statistics, McDonald's ω reliability values, and Pearson product-moment correlation coefficients were also computed.

A multivariate analysis of variance (MANOVA) was performed to identify potential differences by gender and type of sport (i.e., individual and team) in the scores of dependent variables. The hypotheses of the study were tested using path analysis conducted in *M*plus [v. 8.5; (51)]. The hypothesized model is shown in Figure 1. The parameters were estimated using the robust maximum likelihood estimator (MLR) for non-normal data. An acceptable model fit was inferred through multiple criteria: a normed chi-square (*χ*^2^/df) below 5; comparative fit index (CFI) and a Tucker-Lewis index (TLI) values close to 0.95; and root mean square error of approximation (RMSEA) and standardized root mean square residual (SRMR) below .06 (52). Indirect effects in the path model were assessed via maximum likelihood estimator (ML) and a bias-corrected bootstrap method with 5,000 resamples, generating 95% confidence intervals (CIs) around standardized estimates (*β*), with significance indicated by a CI excluding zero (53). Bootstrapping is a method used to accurately calculate CI without making strong assumptions about the underlying distribution of the data (42).

Path analysis was also conducted on the general Psychological, Biological, and Social components of Psychobiosocial experiences (see Measures section) to examine possible differential effects of mindfulness and cognitive appraisals on these components. Exploratory structural equation modeling [ESEM; (54, 55)] was initially performed to determine whether the three components of Psychobiosocial experiences could be represented. The ESEM model was estimated using Target rotation, which, similarly to the confirmatory factor analysis (CFA) approach, relies on the *a priori* specification of the key construct indicators with all cross-loadings being freely estimated but with a target value close to zero. The robust maximum likelihood estimator (MLR) for non-normal data was used.

## 3 Results

Using Mahalanobis distance (*p* < .001), four multivariate outliers were identified and excluded from further analysis. There were no missing data. Descriptive statistics, correlation coefficients, and Mcdonald's *ω* values are reported in Table 1. MANOVA yielded significant differences by gender, Wilks' *λ* = .866, *F*(11, 320) = 4.484, *p* < .001, *η*_p_^2^ = 0.134, sport, Wilks' *λ* = .911, *F*(11, 320) = 2.859, *p* = .001, *η*_p_^2^ = 0.089, and gender × sport interaction, Wilks' *λ* = .921, *F*(11, 320) = 2.482, *p* = .005, *η*_p_^2^ = 0.079. Univariate follow-up tests at *p* ≤ .002 showed that Anger scores were higher in men than in women, and that Anxiety scores were higher in individual sports than in team sports. Furthermore, the highest Anxiety scores were found in women from individual sports. Finally, Challenge appraisal scores were higher in men. Thus, gender, sport, and their interaction were entered as covariate in path analysis.

**Table 1:** Descriptive statistics, Pearson product-moment correlation coefficients, and mcdonald's Omega (*ω*) values.

	Men (*n* = 188)		Women (*n* = 146)														
	*M*	*SD*	*M*	*SD*	Skeweness	Kurtosis	1	2	3	4	5	6	7	8	9	10	*ω*
1. Mindful awareness	4.60	0.58	4.60	0.60	0.18	−0.35	–										.604
2. Non-judgmental attitude	3.12	1.07	2.87	0.98	0.42	0.06	−.37^a^	–									.713
3. Refocusing	4.55	0.70	4.46	0.62	−0.28	0.03	.45^b^	−.11	–								.715
4. Challenge appraisal	5.23	0.62	4.95	0.87	−0.71	0.18	.36^a^	−.06	.35^a^	–							.759
5. Threat appraisal	1.56	0.80	1.60	0.76	1.44	1.55	−.29^a^	.02	−.26^a^	−.44^b^	–						.876
6. Excitement	2.62	0.92	2.73	0.82	−0.49	0.09	.23^a^	−.14	.22^a^	.42^b^	−.24^a^	–					–
7. Happiness	2.67	1.00	2.68	0.88	−0.23	−0.85	.25^a^	−.10	.23^a^	.50^b^	−.34^a^	.67^c^	–				–
8. Anxiety	1.25	0.88	1.52	1.01	0.50	−0.15	−.07	−.17	−.24^a^	−.36^a^	.35^a^	−.02	−.17	–			–
9. Dejection	0.36	0.57	0.44	0.72	1.66	2.58	−.12	−.12	−.16	−.40^b^	.45^b^	−.13	−.26^a^	.37^a^	–		–
10. Anger	0.48	0.71	0.25	0.52	1.62	1.87	−.09	−.06	−.14	−.12	.27^a^	−.01	−.22^a^	.25^a^	.55^b^	–	–
11. Psychobiosocial experiences	2.01	0.90	1.95	0.94	−0.57	0.26	.32^a^	−.08	.50^b^	.59^b^	−.44^b^	.53^b^	.55^b^	−.26^a^	−.38^a^	−.20^a^	.886

Correlation.

^a^ Low.

^b^ Moderate.

^c^ Moderately high (56). Excitement, happiness, anxiety, dejection, and Anger were single-item measures.

In the entire sample, the mean scores of Challenge appraisal were higher than those of Threat appraisal. Similarly, the mean scores of pleasant emotions (Excitement and Happiness) were higher than those of unpleasant emotions (Anxiety, Dejection, and Anger). All differences were significant at *p* < .001. These results, along with the positive mean scores of Psychobiosocial experiences, suggest that the athletes in this sample perceived upcoming competitions as more challenging, pleasant, and functional rather than threatening, unpleasant, and dysfunctional. Correlation analysis (Table 1) showed that both Awareness and Refocusing were related positively to Challenge appraisal, Excitement, Happiness, and Psychobiosocial experiences, and negatively to Threat appraisal. Challenge appraisal was related positively to Excitement, Happiness, and Functional experiences, and negatively to Anxiety and Dejection, while Threat appraisal was associated negatively with Excitement, Happiness, and Psychobiosocial experiences, and positively with Anxiety, Dejection, and Anger.

**Figure 2:**
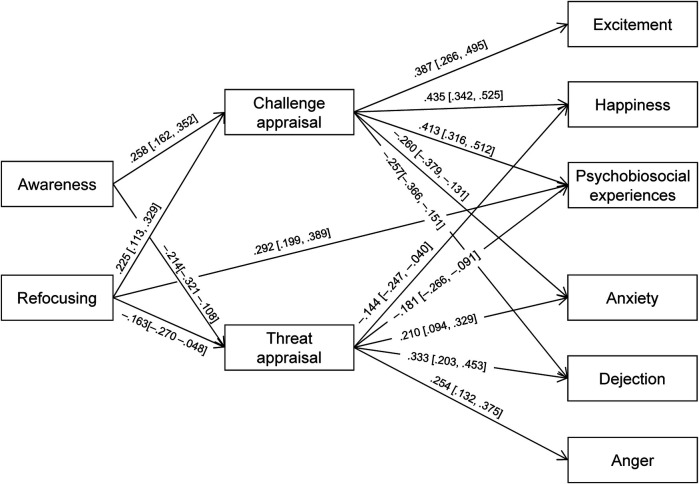
Path analysis results of the relationships between mindfulness (awareness and refocusing) and cognitive appraisals (challenge and threat) on pleasant emotions (excitement and happiness), psychobiosocial experiences, and unpleasant emotions (anxiety, dejection, and anger). All standardized values (*β*) are significant at *p* < .05 (95% CI are in square brackets). Only significant paths are presented.

Path analysis, controlling for gender, sport, and their interaction, was conducted to examine the hypothesized relationships represented in Figure 1. Non-judgmental attitude was not included in the analysis because it did not correlate with any of the other variables. The model did not fit the data well, *χ*^2^/df = 3.546, CFI = .926, TLI = .843, RMSEA = .087 (90% CI = .070–.105), SRMR = .048. Inspection of modification indices suggested fit improvement after adding one path in the model from Refocusing to Psychobiosocial experiences. This addition is in line with theoretical assumptions. The revised model depicted in Figure 2 provided adequate fit, *χ*^2^/df = 1.915, CFI = .974, TLI = .943, RMSEA = .052 (90% CI = .032–.072), SRMR = .038. All predicted paths were significant, except the anticipated negative links between Challenge appraisal and Anger, and between Threat appraisal and Excitement. As expected, bootstrap analysis resulted in significant indirect effects from mindfulness to the outcome variables. As shown in Table 2, all indirect effects were significant, with the exception of the paths from Awareness/Refocusing to Excitement through Threat appraisal, and from Awareness/Refocusing to Anger via Challenge appraisal. Therefore, the two hypotheses of the study were substantially confirmed.

**Table 2:** Indirect effects results for paths from mindfulness (awareness and refocusing) to pleasant emotions (excitement and happiness), psychobiosocial experiences, and unpleasant emotions (anxiety, dejection, and anger) via cognitive appraisals (challenge and threat).

Effect	*β*	SE	Bootstrap bias–corrected 95% CI (lower, upper)	
Total indirect Awareness → Excitement	.115*	.025	.070	.166
Awareness → Challenge → Excitement	.100*	.026	.054	.157
Awareness → Threat → Excitement	.015	.014	−.007	.051
Total indirect Refocusing → Excitement	.098*	.027	.049	.154
Refocusing → Challenge → Excitement	.087*	.025	.044	.144
Refocusing → Threat → Excitement	.011	.011	−.004	.040
Total indirect Awareness → Happiness	.143*	.029	.091	.202
Awareness → Challenge → Happiness	.112*	.026	.067	.168
Awareness → Threat → Happiness	.031*	.015	.007	.068
Total indirect Refocusing → Happiness	.121*	.031	.062	.186
Refocusing → Challenge → Happiness	.098*	.026	.049	.153
Refocusing → Threat → Happiness	.024*	.012	.005	.054
Total indirect Awareness → Psychobiosocial	.145*	.031	.087	.210
Awareness → Challenge → Psychobiosocial	.107*	.027	.060	.165
Awareness → Threat → Psychobiosocial	.039*	.014	.018	.072
Total indirect Refocusing → Psychobiosocial	.122*	.029	.065	.182
Refocusing → Challenge → Psychobiosocial	.093*	.024	.048	.143
Refocusing → Threat → Psychobiosocial	.029*	.013	.008	.061
Total indirect Awareness → Anxiety	−.112*	.021	−.158	−.074
Awareness → Challenge → Anxiety	−.067*	.017	−.106	−.038
Awareness → Threat → Anxiety	−.045*	.015	−.082	−.021
Total indirect Refocusing → Anxiety	−.093*	.029	−.152	−.039
Refocusing → Challenge → Anxiety	−.058*	.024	−.113	−.020
Refocusing → Threat → Anxiety	−.034*	.017	−.077	−.008
Total indirect Awareness → Dejection	−.137*	.028	−.195	−.085
Awareness → Challenge → Dejection	−.066*	.021	−.115	−.032
Awareness → Threat → Dejection	−.071*	.020	−.116	−.037
Total indirect Refocusing → Dejection	−.112*	.031	−.176	−.053
Refocusing → Challenge → Dejection	−.058*	.018	−.101	−.028
Refocusing → Threat → Dejection	−.054*	.023	−.106	−.016
Total indirect Awareness → Anger	−.066*	.021	−.112	−.029
Awareness → Challenge → Anger	−.012	.018	−.052	.020
Awareness → Threat → Anger	.054*	.018	−.099	−.026
Total indirect Refocusing → Anger	−.052*	.021	−.098	−.015
Refocusing → Challenge → Anger	−.010	.016	−.046	.018
Refocusing → Threat → Anger	−.041*	.019	−.086	−.011

*β,* standardized estimate; SE, standard error; CI, confidence interval.

* Significance indicated via 95% CI.

**Figure 3:**
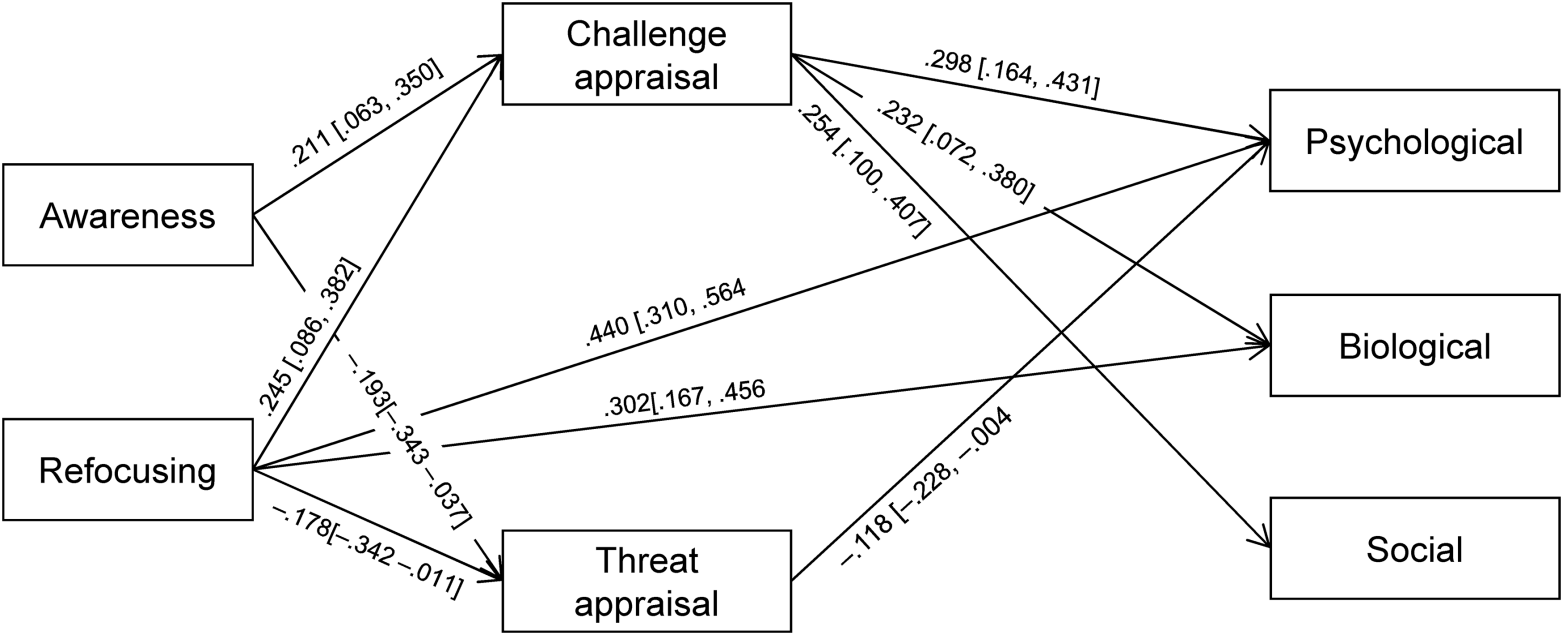
Path analysis results of the relationships between mindfulness (awareness and refocusing) and cognitive appraisals (challenge and threat) on psychological, biological, and social components of psychobiosocial experiences. All standardized values (*β*) are significant at *p* < .05 (95% CI are in square brackets). The Psychological component includes emotion, confidence, anxiety, assertiveness, cognitive, motivational, and volitional items. The Biological component includes bodily-somatic, motor-behavioral, and operational items. The Social component includes communicative and social support items. Only significant paths are presented.

ESEM on the three components of Psychobiosocial experiences yielded an acceptable fit to the data, *χ*^2^/df = 2.578, CFI = .956, TLI = .913, RMSEA = .069 (90% CI = .051–.087), SRMR = .030. Therefore, mean scores of Psychological, Biological, and Social components were entered in path analysis with mindfulness and cognitive appraisals acting as antecedents and mediators, respectively. The model resulted in a poor fit to the data, *χ*^2^/df = 4.897, CFI = .862, TLI = .654, RMSEA = .155 (90% CI = .113–.198), SRMR = .101. Fit substantially improved with the addition of two paths from Refocusing to the Psychological and Biological components, *χ*^2^/df = 1.107, CFI = .997, TLI = .991, RMSEA = .025 (90% CI = .000–.097), SRMR = .047. The final model is shown in Figure 3. Bootstrap analysis yielded significant indirect effects from mindfulness to the Psychobiosocial components. As shown in Table 3, all indirect effects were significant, except for the paths from Awareness/Refocusing to the Biological component via Threat appraisal, and from Refocusing to the Social component via Threat appraisal. Thus, findings provided full support for Hypothesis 1 and partial support for Hypothesis 2 limited to the Psychological component of Psychobiosocial experiences.

**Table 3:** Indirect effects results for paths from mindfulness (awareness and refocusing) to psychological, biological, and social components of psychobiosocial experiences.

Effect	*β*	SE	Bootstrap bias–corrected 95% CI (lower, upper)	
Total indirect Awareness → Psychological	.086*	.033	.030	.162
Awareness → Challenge → Psychological	.063*	.030	.017	.134
Awareness → Threat → Psychological	.023*	.014	.003	.061
Total indirect Refocusing → Psychological	.094*	.031	.039	.163
Refocusing → Challenge → Psychological	.073*	.028	.028	.140
Refocusing → Threat → Psychological	.021*	.014	.002	.063
Total indirect Awareness → Biological	.067*	.033	.017	.143
Awareness → Challenge → Biological	.049*	.027	.010	.115
Awareness → Threat → Biological	.018	.018	–.006	.072
Total indirect Refocusing → Biological	.074*	.031	.024	.148
Refocusing → Challenge → Biological	.057*	.027	.015	.124
Refocusing → Threat → Biological	.017	.019	–.005	.077
Total indirect Awareness → Social	.086*	.035	.029	.165
Awareness → Challenge → Social	.054*	.026	.014	.117
Awareness → Threat → Social	.033*	.024	.001	.103
Total indirect Refocusing → Social	.092*	.038	.030	.179
Refocusing → Challenge → Social	.062*	.030	.017	.141
Refocusing → Threat → Social	.030	.024	–.001	.100

*β,* standardized estimate; SE, Standard error; CI, Confidence interval. The Psychological component includes emotion, confidence, anxiety, assertiveness, cognitive, motivational, and volitional items. The Biological component includes bodily-somatic, motor-behavioral, and operational items. The Social component includes communicative and social support items.

* Significance indicated via 95% CI.

## 4 Discussion

The current study examined the effect of mindfulness and cognitive appraisals on emotional experiences in athletes. Study hypotheses were rooted in MuSt theory (34), as well as the transactional model of stress (25) and the theory of challenge and threat states in athletes (28, 29).

### 4.1 Mindfulness, cognitive appraisals, and emotional experiences

The study findings highlight the positive relationship between dispositional mindfulness and the experience of pleasant emotions in athletes, aligning with existing literature that underscores the critical role of mindfulness in improving emotional and psychobiosocial experiences (22, 24). Mindfulness, which involves cultivating awareness and acceptance of the present moment, enables athletes to observe their thoughts and feelings without reacting to or altering their emotional responses (8). This mindful awareness is expected to help athletes refocus on their tasks and engage with their emotions constructively, reducing the likelihood of being overwhelmed by negative feelings that could hinder performance. According to MuSt theory (34), mindfulness facilitates effective self-regulation, allowing athletes to achieve and maintain an optimal performance state. Moreover, the results of the study suggest that cognitive appraisals play a significant role in emotional experiences, with high levels of challenge appraisal—where competition is viewed as an opportunity to demonstrate one's potential—being linked to more pleasant and functional emotional experiences. Conversely, high levels of threat appraisal—where competition is seen as overwhelming—are associated with more unpleasant emotions. These findings support the notion that athletes who successfully engage in challenge appraisal experience enhanced emotional states and psychophysical functioning, consistent with existing research [e.g., (30, 31); see (5)].

It is worth noting that the non-judgmental attitude component of dispositional mindfulness did not correlate with the other variables in the study (i.e., cognitive appraisals, emotions, and psychobiosocial experiences). In the competitive context, athletes are often conditioned to adopt an evaluative mindset, where constant assessment of their performance, strengths, and weaknesses is fundamental for success. This performance-oriented approach might not align well with the non-judgmental attitude, which involves accepting thoughts and emotions without criticism. During high-pressure situations, such as competitions, athletes may spontaneously prioritize a mindfulness mindset that enhances awareness and focus on the task over non-judgmental acceptance. Consequently, non-judgmental attitude might not directly impact their immediate cognitive appraisals, emotional states, or psychobiosocial experiences in the same way that other aspects of mindfulness do. Moreover, the non-judgmental attitude might require a deeper level of mindfulness practice, which some athletes may not have fully developed.

### 4.2 The mediating role of cognitive appraisals

According to the main purposes of the study, the first hypothesis proposed that challenge appraisal would positively mediate the relationship between mindfulness and pleasant emotions/psychobiosocial experiences, while negatively mediating the relationship with unpleasant emotions. The findings largely supported this hypothesis. Path analysis revealed that mindfulness was indeed positively associated with challenge appraisal, which, in turn, was positively related to pleasant emotions and functional psychobiosocial experiences. Significant indirect effects from mindfulness to most of these variables, except for anger, through challenge appraisal further substantiated this relationship. The results suggest that higher levels of mindfulness enhance challenge appraisal, which subsequently increase pleasant and functional experiences and reduce unpleasant emotions.

Regarding the psychobiosocial experience components (i.e., psychological, biological, and social), findings revealed that challenge appraisal positively mediated the relationship between mindfulness and all three components. Again, these results support the first hypothesis, indicating that mindfulness enhances the athletes' ability to appraise situations as challenges, which in turn positively influences their psychological, biological, and social experiences. This result concurs with the notion that mindfulness promotes an adaptive mindset, enabling athletes to perceive potentially stressful situations as opportunities for growth and improved performance. Accordingly, it might be suggested that by fostering challenge appraisal, mindfulness may enhance not only emotional, cognitive, and motivational aspects that are incorporated in the psychological component, but also improve bodily (e.g., feeling energetic and reactive) and motor-behavioral aspects (e.g., feeling coordinated and skillful) included in the biological component, and social interactions aspects (e.g., feeling communicative and supported) represented in the social component. This aligns with MuSt theory (34), which emphasizes the role of mindfulness in improving psychological flexibility and a more adaptive approach to emotional regulation. According to MuSt theory, mindfulness promotes a mindset characterized by mindful awareness of the own feelings and refocused attention on the performance-related task, enabling athletes to perceive challenging situations more positively.

The second hypothesis proposed that threat appraisal would negatively mediate the relationship between mindfulness and pleasant emotions/psychobiosocial experiences, while positively mediating the relationship with unpleasant emotions. The findings provided partial support for this hypothesis. Mindfulness was negatively linked to threat appraisal, which was positively associated with unpleasant emotions and negatively related to pleasant emotions/psychobiosocial experiences. However, not all expected indirect paths were significant. Specifically, the indirect relationships between awareness/refocusing and excitement through threat appraisal, and between awareness/refocusing and anger via challenge appraisal were not significant. As for the significant indirect effects, the results suggest that higher mindfulness levels are linked to lower threat appraisal levels, thereby reducing the negative effects of threat appraisal on pleasant and functional experiences and mitigating its harmful enhancement of unpleasant emotions.

Significant indirect effects were also observed for the psychological component of psychobiosocial experiences, thereby suggesting that mindful awareness and refocusing positively influence emotional, cognitive, and motivational feelings not only through enhanced challenge appraisal, but also through reduced threat appraisal. However, this mediating effect of threat appraisal was not significant when referred to the biological and social components. The lack of significant indirect effects may be due to the overall low scores of threat appraisal (in-between “*totally disagree*” to “*rather disagree*”) in the sample, which were substantially lower than challenge appraisal scores. This is also reflected on the mean scores of pleasant emotions that were significantly higher than those of unpleasant emotions. It is suggested that the athletes in our sample tended to view competition as a more exciting and less threatening opportunity to express their skills and achieve success, likely believing they had the necessary resources and support to meet the competitive demands of their sport.

### 4.3 Limitations and future research directions

Some limitations in the study should be considered for future research. The cross-sectional design, while informative, prevents definitive conclusions about cause-and-effect relationships between mindfulness, cognitive appraisals, and emotional experiences. Future research could address this issue using longitudinal or experimental designs to investigate the dynamic interplay of these factors over time. Another issue is the reliance on self-reported data, which is inherently susceptible to biases like social desirability and recall errors. Triangulating findings with objective measures, such as physiological assessments or behavioral observations, would strengthen the validity of future studies. Furthermore, incorporating performance and well-being measures, as posited in MuSt theory (34), would contribute to our understanding of the direct and indirect effects of mindfulness, cognitive appraisals, and emotional experiences on these variables. Also, expanding research to include action components alongside emotional responses, as emphasized in MuSt theory (57), would provide a more comprehensive view of the antecedents and mediators of emotions and performance. Finally, exploring the impact of demographic factors (age, gender, experience level) and examining a wider range of antecedents like resilience (58), self-compassion (59), and organizational factors (60) would provide a more nuanced representation of the complex interactions of variables. Within the MuSt theory framework, future research could also investigate the efficacy of tailored mindfulness interventions for athletes, focusing on how these programs influence cognitive appraisal and emotional regulation strategies to optimize performance.

### 4.4 Practical implications

Coaches and sport psychologists can guide athletes to adopt a challenge-oriented mindset, viewing competitions as opportunities for growth rather than threats. This perspective emphasizes developing mindful awareness and refocusing attention, skills that can be enhanced through mindfulness-based interventions (12, 14). By embracing challenges and focusing on the present moment, athletes can cultivate a sense of optimism and resilience. Mindfulness techniques, such as body awareness exercises, mindful breathing, and yoga (6, 61), should be integrated into training routines to foster mindful attitudes. Athletes should engage in continuous self-reflection, regularly observing their cognitive appraisals and emotional experiences. This process helps them identify and manage stressors, develop problem-solving skills, and regulate their behaviors and emotional responses. Through self-reflection, athletes gain a deeper understanding of their mental processes, leading to improved control and adaptability in approaching competitions. For mindfulness interventions to be successful, a supportive environment that encourages emotional expression and adaptive emotion regulation is crucial (62). This environment promotes a sense of security and confidence, allowing athletes to explore their emotions and develop functional self-regulation.

### 4.5 Conclusion

The study supports the notion that dispositional mindfulness influences emotional experiences through its effects on cognitive appraisals. In particular, findings suggest that the influence of mindfulness on both challenge and threat appraisals leads to enhanced pleasant and functional experiences and reduced unpleasant emotions. These results reinforce the importance of mindfulness and cognitive appraisal processes in emotional regulation. Overall findings support the MuSt theory underpinnings and offer practical guidance for coaches and sport psychologists to optimize performance and enhance well-being of athletes.

## Data Availability

The raw data supporting the conclusions of this article will be made available by the authors, without undue reservation.

## Supplementary material

Supplementary Data Sheet 1 Performance-related psychobiosocial experiences.
