# Supplementary material for: Cognitive appraisals linking dispositional mindfulness to athletes' emotions: a multi-states theory approach
Source: Front Sports Act Living. 2024 Dec 18;6:1521613. doi: 10.3389/fspor.2024.1521613 (PMC11688466; doi:10.3389/fspor.2024.1521613)
Supplement: Supplementary Data Sheet 1 — Performance-related psychobiosocial experiences. [file Datasheet1.pdf]

**Cognitive Appraisals Linking Dispositional Mindfulness to Athletes' Emotions:
A Multi-States Theory Approach**

Supplementary Material

Performance-related Psychobiosocial Experiences

Below, you will find a list of adjectives (descriptors) commonly used by athletes to describe their feelings about their performance. Each row presents opposing descriptors, with some being potentially dysfunctional (left side) and others functional (right side) for performance. Read them carefully and for each row reflect on one or more descriptors, either **on the left or on the right**, which best reflect **how you feel in relation to your upcoming major competition**. Then, rate the intensity of the chosen descriptor(s) on the scale ranging from 1 (***little***) to 4 (***very much***). If none of the descriptors in a particular row reflect how you feel regarding the competition, please select the middle box 0 (***neither… nor***). There are no right or wrong answers. Please, make sure to complete all rows.

Example:
"I feel moderately satisfied (gratified, pleased, fulfilled) with myself". In this case check box 2 on the right side.

dissatisfied, disappointed, displeased, discontented	4	3	2	1	0	1	~~2~~ (X)	3	4	satisfied, gratified, pleased, fulfilled

On the other hand, if for you it is true: "I feel much dissatisfied (disappointed, displeased, discontented) with myself", then check box 3 on the left side.

dissatisfied, disappointed, displeased, discontented	4	~~3~~ (X)	2	1	0	1	2	3	4	satisfied, gratified, pleased, fulfilled

		Very much	Much	Moderate	Little	neither… nor	Little	Moderate	Much	Very much	
1	dejected, unhappy, sad, distressed, upset	4	3	2	1	0	1	2	3	4	enthusiastic, happy, joyful, cheerful
2	unconfident, incapable insecure, uncertain	4	3	2	1	0	1	2	3	4	confident, capable, secure, certain
3	anxious, worried, tense, nervous in a harmful way	4	3	2	1	0	1	2	3	4	anxious, worried, tense, nervous in a helpful way
4	timid, fragile, reserved, surrendered	4	3	2	1	0	1	2	3	4	fighting spirit, fierce, aggressive, combative
5	distracted, off-task, inattentive, unfocused	4	3	2	1	0	1	2	3	4	alert, on-task, attentive, focused
6	unmotivated, disengaged, uninterested, uninspired	4	3	2	1	0	1	2	3	4	motivated, engaged, interested, inspired
7	hesitant, indecisive, undetermined, irresolute	4	3	2	1	0	1	2	3	4	purposeful, decisive, persistent, determined
8	physically fatigued, tired, drained, drowsy	4	3	2	1	0	1	2	3	4	physically vigorous, charged, reactive, energetic
9	sluggish, uncoordinated, clumsy, weak movement	4	3	2	1	0	1	2	3	4	active, coordinated, dynamic, smooth movement
10	ineffective, unskillful, unstable, inconsistent performance	4	3	2	1	0	1	2	3	4	effective, skillful, stable, consistent performance
11	uncommunicative, withdrawn, disconnected, detached	4	3	2	1	0	1	2	3	4	communicative, sociable, expansive in a helpful way
12	abandoned, ignored, neglected, rejected	4	3	2	1	0	1	2	3	4	assisted, considered, supported, accepted

Scoring

Scores on the dysfunctional side (i.e., left side) are transformed into negative scores. Thus, the score of an item could range from –4 to 4.

The Psychological component includes emotion, confidence, anxiety, assertiveness, cognitive, motivational, and volitional items: 1, 2, 3, 4, 5, 6, 7.

The Biological component includes bodily-somatic, motor-behavioral, and operational items: 8, 9, 10.

The Social component includes communicative and social support items: 11, 12.

Note: The English version here presented is a translation of the Italian version (see next page) and has not been validated.

Esperienze Psicobiosociali Associate alla Prestazione

Di seguito è riportato un elenco di aggettivi (descrittori) comunemente utilizzati dagli atleti per descrivere come si sentono in relazione alla loro prestazione. Ogni riga presenta descrittori opposti, alcuni potenzialmente disfunzionali (lato sinistro) e altri funzionali (lato destro) per la prestazione. Leggili attentamente e per ogni riga rifletti su uno o più descrittori, situati **nella colonna a sinistra o nella colonna a destra**, che descrivono **come ti senti in relazione alla tua prossima competizione importante**. Quindi, valuta l'intensità del/i descrittore/i scelto/i su una scala da 1 (***un po'***) a 4 (***moltissimo***). Se nessuno dei descrittori in una particolare riga riflette la tua esperienza competitiva, seleziona la casella centrale 0 (***né... né***). Non ci sono risposte giuste o sbagliate. Assicurati di completare tutte le righe.

Esempio:
"Mi sento abbastanza soddisfatto (gratificato, contento, appagato) di me stesso". In questo caso seleziona la casella 2 sul lato destro.

insoddisfatto, frustrato, scontento, deluso	4	3	2	1	0	1	~~2~~ (X)	3	4	soddisfatto, gratificato, contento, appagato

D'altra parte, se per te è vero: "Mi sento molto insoddisfatto (frustrato, scontento, deluso) di me stesso", allora seleziona la casella 3 sul lato sinistro.

insoddisfatto, frustrato, scontento, deluso	4	~~3~~ (X)	2	1	0	1	2	3	4	soddisfatto, gratificato, contento, appagato

		Moltissimo	Molto	Abbastanza	Un po'	Né...né	Un po'	Abbastanza	Molto	Moltissimo	
1	demoralizzato, infelice, triste, avvilito	4	3	2	1	0	1	2	3	4	entusiasta, felice, gioioso, allegro
2	sfiduciato, incapace, insicuro, incerto	4	3	2	1	0	1	2	3	4	fiducioso, capace, sicuro, certo
3	ansioso, preoccupato, teso, nervoso in modo dannoso	4	3	2	1	0	1	2	3	4	ansioso, preoccupato, teso, nervoso in modo utile
4	remissivo, fragile, docile, arrendevole	4	3	2	1	0	1	2	3	4	combattivo, grintoso, aggressivo, agguerrito
5	distratto, deconcentrato, disattento, svagato	4	3	2	1	0	1	2	3	4	vigile, concentrato, attento, focalizzato
6	demotivato, disimpegnato, disinteressato, indifferente	4	3	2	1	0	1	2	3	4	motivato, coinvolto, interessato, appassionato
7	titubante, indeciso, rinunciatario, incostante, esitante	4	3	2	1	0	1	2	3	4	risoluto, convinto, tenace, perseverante, determinato
8	fisicamente debole, scarico, non reattivo, affaticato	4	3	2	1	0	1	2	3	4	fisicamente vigoroso, carico, reattivo, energico
9	movimento fiacco, scoordinato, impacciato, goffo	4	3	2	1	0	1	2	3	4	movimento attivo, coordinato, dinamico, fluido
10	prestazione inefficace, scadente, instabile	4	3	2	1	0	1	2	3	4	prestazione efficace, abile, stabile
11	non comunicativo, isolato, sconnesso, distaccato	4	3	2	1	0	1	2	3	4	comunicativo, espansivo, socievole in modo utile
12	abbandonato, ignorato, trascurato, rifiutato	4	3	2	1	0	1	2	3	4	assistito, considerato, supportato, accettato
